# Oxidative status and intestinal health of gilthead sea bream (*Sparus aurata*) juveniles fed diets with different ARA/EPA/DHA ratios

**DOI:** 10.1038/s41598-020-70716-5

**Published:** 2020-08-14

**Authors:** R. Magalhães, I. Guerreiro, R. A. Santos, F. Coutinho, A. Couto, C. R. Serra, R. E. Olsen, H. Peres, A. Oliva-Teles

**Affiliations:** 1 CIMAR/CIIMAR – Centro Interdisciplinar de Investigação Marinha e Ambiental, Terminal de Cruzeiros do Porto de Leixões, Universidade do Porto, Av. General Norton de Matos, 4450-208 Matosinhos, Portugal; 2 Departamento de Biologia, Faculdade de Ciências, Universidade do Porto, Rua do Campo Alegre, Edifício FC4, 4169-007 Porto, Portugal; 3 Department of Biology, Norwegian University of Science and Technology, Trondheim, Norway

**Keywords:** Microbiology, Physiology, Zoology

## Abstract

The present work assessed the effects of dietary ratios of essential fatty acids, arachidonic (ARA), eicosapentaenoic (EPA) and docosahexaenoic acid (DHA), on liver and intestine oxidative status, intestinal histomorphology and gut microbiota of gilthead sea bream. Four isoproteic and isolipidic plant-based diets were formulated containing a vegetable oil blend as the main lipid source. Diets were supplemented with ARA/EPA/DHA levels (%DM) equivalent to: 2%:0.2%:0.1% (Diet A); 1.0%:0.4%:0.4% (Diet B); 0%:0.6%:0.6% (Diet C); 0%:0.3%:1.5% (Diet D) and tested in triplicate groups for 56 days. Lipid peroxidation was higher in fish fed diets C and D while no differences were reported between diets regarding total, oxidized, and reduced glutathione, and oxidative stress index. Glutathione reductase was higher in fish fed diet A than diets C and D. No histological alterations were observed in the distal intestine. Lower microbiota diversity was observed in intestinal mucosa of fish fed diet C than A, while diets C and D enabled the proliferation of health-promoting bacteria from Bacteroidetes phylum (*Asinibacterium* sp.) and the absence of pathogenic species like *Edwardsiella tarda*. Overall, results suggest that a balance between dietary ARA/EPA + DHA promotes gilthead sea bream juveniles’ health however higher dietary content of n-3 LC-PUFA might limited the presence of microbial pathogens in intestinal mucosa.

## Introduction

In marine fish species, dietary supply of linoleic (LA; 18:2n-6) and α-linolenic (ALA; 18:3n-3) acids cannot meet essential fatty acids (EFA) requirements due to the evolutionary loss or low capacity of long-chain polyunsaturated fatty acids (LC-PUFA) biosynthesis^1–3^. In gilthead sea bream (*Sparus aurata*), the leading finfish species in Mediterranean aquaculture, the limited capacity to synthesize LC-PUFAs from C-18 precursors is related to low activities of the Δ5 fatty acid desaturase and elongase 2^3,4^. Thus, dietary supply of the main LC-PUFA, arachidonic acid (ARA; 20:4n-6), eicosapentaenoic acid (EPA; 20:5n-3), and docosahexaenoic acid (DHA; 20:6 n-3) is necessary to fulfill the physiological requirements of EFA of this species^3,5^.

For gilthead sea bream juveniles, total dietary EPA + DHA requirements vary with lipid level and DHA/EPA ratio and were estimated to be 0.9 or 1.9% with a DHA/EPA ratio of 1 or 0.5, respectively^6–8^. However individual EPA and DHA requirements have been reported to be 0.7 and 0.6% of dry matter (DM), respectively (reviewed by^9^). As for ARA, the only available study indicates that increasing the dietary levels (0, 0.6, 1.14, 1.7%DM) has no effect on growth performance^10^. However, in European sea bass juveniles, dietary ARA levels below 0.2% reduced growth performance^11^.

In marine aquafeeds, the main source of LC-PUFA has been fish oil (FO) but, due to its stagnant availability in the world market, its dietary replacement by alternative lipid sources is required for promoting further growth of the marine aquaculture industry.

Thus, in recent years, research focused on FO replacement by more available and sustainable oil sources, such as vegetable oils (VO)^12–15^, land animal oils^13,16–18^, or microalgae oils^19–21^. However, most of the commercially available alternative oil sources, namely VO and animal oils, are deficient in LC-PUFA and its dietary inclusion may jeopardize normal fish growth, health, intestinal histomorphology, and microbiota community^5,22–24^.

LC-PUFAs are highly susceptible to peroxidation, generating reactive oxygen species (ROS) causing tissue oxidative damage^14,25,26^.

ROS induce damage to lipids, proteins, carbohydrates, and nucleic acids, with the potential to induce cellular death and compromising tissue functionality and palatability of the final product^14,25^. LC-PUFA oxidation can compromise membrane structure, by decreasing its fluidity and increasing its permeability to harmful substances, with potential to be pathological to the cell or tissue^27^. The balance between generation and clearance of ROS is maintained through the action of the antioxidant system, involving radical scavenging enzymes (catalase, CAT; superoxide dismutase, SOD; glutathione reductase, GR; and glutathione peroxidase, GPX) and non-enzymatic antioxidants (Vitamin A, C, and E; glutathione, GSH; bilirubin and flavonoids)^25,28^. However, this balance may be highly affected by diet composition, including the level of LC-PUFA. For instance, increasing dietary levels of n-3 LC-PUFA were associated with increased liver and plasma oxidative stress of silvery black porgy (*Sparidentex hasta*)^29^. Also, high dietary content of n-3 LC-PUFA increased oxidative stress in the muscle of rainbow trout, *Oncorhynchus mykiss*^30^, the intestine of European sea bass (*Dicentrarchus labrax*)^31^ and liver and intestine of gilthead sea bream^14^. Increasing levels of ARA were also shown to increase liver oxidative stress in *Synechogobius hasta* juveniles^32^ and Japanese eel serum (*Anguilla japonica*)^33^.

Dietary DHA/EPA ratio also seems to affect oxidative stress, apparently in a species-specific manner. For instance, higher ratios caused increased oxidative damage in serum of black sea bream (*Acanthopagrus schlegelii*) while the opposite was observed in the plasma of silvery-black porgy juveniles^2,34^. In an in vitro study, it was shown that increasing concentrations of EPA, but not of DHA, increased lipid peroxidation levels in yellow croaker (*Larmichthys crocea*) macrophages^35^. Further, while EPA increased superoxide anion production the opposite was observed for DHA. Thus, EPA and DHA seem to have different capacities for modulating redox mechanisms.

Intestinal microbiota is vital to promote fish physiological balance and health, and its effects on fish feeding, digestion, metabolism, immunity, and disease resistance have been highlighted^24,36,37^. Fish intestinal microbiota is modulated by several factors including host genetic background, living environment, section of the gastrointestinal tract, stress, and diet^24,36,37^. Intestinal microbiota seems to be particularly sensitive to diet composition^12,24,38,39^. However, the impact of dietary PUFA, particularly dietary LC-PUFAs, on gut microbiota has been poorly explored booth in humans and aquatic species^12,24,40^.

Previously, Magalhães et al.^41^ showed that a diet with a balanced ARA/EPA/DHA (1/0.4/0.4) ratio that closely matches the estimated EFA requirements of gilthead seabream increased feed efficiency and protein efficiency ratio compared to diets with unbalanced ARA/EPA/DHA ratios (0/0.6/0.6 and 0/0.3/1.5). Dietary ARA/EPA/DHA ratios also affected lipid metabolism, with increased β-oxidation activity in fish fed high ARA/EPA/DHA (2.0/0.2/0.1) and decreased lipogenesis in fish fed high DHA (0/0.3/1.5). Furthermore, liver lipid content was higher in fish fed high dietary EPA and DHA levels.

The objective of this study was to further evaluate dietary ARA/EPA/DHA ratios of the fore mentioned diets on oxidative stress in target organs (liver and intestine), distal intestine histomorphology, and gut microbiota composition of gilthead sea bream juveniles. Therefore, the importance of dietary ARA or n-3 LC-PUFA on these indicators of wellbeing was compared to the EFA balanced diet.

## Results

The results of the feeding trial are presented elsewhere^41^ as it was not the aim of this study.

### Oxidative stress

Oxidative stress indicators, namely liver and intestine enzymatic activities and glutathione responded similarly to the diet treatments, as no organ × diet interaction in any parameter analyzed was observed (Tables 1 and 2).

**Table 1:** Liver and intestine total glutathione (tGSH), oxidized glutathione (GSSG), reduced glutathione (GSH), oxidative stress index (OSI) and lipid peroxidation (LPO) levels of gilthead sea bream fed the experimental diets.

Organ	Liver					Intestine				
Diets	A	B	C	D	SEM	A	B	C	D	SEM
	2.0/0.2/0.1	1.0/0.4/0.4	0/0.6/0.6	0/0.3/1.5		2.0/0.2/0.1	1.0/0.4/0.4	0/0.6/0.6	0/0.3/1.5	
tGSH	1017	935	928	739	51.8	947	797	884	788	42.8
GSSG	39	29	36	28	3.8	111	114	135	133	7.5
GSH	977	906	893	711	49.0	837	675	748	655	42.3
OSI^1^	7.19	6.12	7.25	7.00	0.5	23.7	27.7	33.9	34.3	2.2
LPO	13.5	14.9	18.7	19.5	0.8	69.6	62.6	90.6	79.2	3.0

	Variance source			Diets						
	Diet	Organ	Interaction	A	B	C	D			
**Two-way Anova**										
tGSH	Ns	Ns	ns	–	–	–	–			
GSSG	Ns	0.000	ns	–	–	–	–			
GSH	Ns	0.043	ns	–	–	–	–			
OSI^1^	Ns	0.000	ns	–	–	–	–			
LPO	0.000	0.000	ns	a	a	b	b			

Values presented as means (n = 9 for liver and n = 6 for intestine)) and pooled standard error of the mean (SEM). LPO values expressed as nmols MDA g^−1^ tissue and GSH, tGSH, and GSSG as nmol g^−1^ tissue

Two-way ANOVA: ns: non-significant (*P* > 0.05).

^1^OSI = 100 ×  (2 × GSSG/tGSH).

**Table 2:** Liver and intestine antioxidant enzymes activity of gilthead sea bream fed the experimental diets.

Organ	Liver					Intestine				
Diets	A	B	C	D	SEM	A	B	C	D	SEM
	2.0/0.2/0.1	1.0/0.4/0.4	0/0.6/0.6	0/0.3/1.5		2.0/0.2/0.1	1.0/0.4/0.4	0/0.6/0.6	0/0.3/1.5	
CAT	56.7	51.8	42.1	48.5	1.68	153.8	92.4	76.6	158.6	16.0
G6PDH	136.6	132.1	127.8	110.0	5.25	24.6	25.8	31.0	20.2	1.66
GR	8.8	7.3	7.0	6.6	0.28	20.7	15.9	14.9	16.1	0.96
SOD	106.9	126.9	110.3	108.5	6.59	1,341.4	1,220.3	1,232.5	929.0	63.7
GPX	54.2	36.4	38.5	35.5	3.22	10.2	9.6	9.6	9.0	0.64

	Variance source			Diets						
	Diet	Organ	Interaction	A	B	C	D			
**Two-way Anova**										
CAT	0.022	0.000	ns	–	–	–	–			
G6PDH	Ns	0.000	ns	–	–	–	–			
GR	0.008	0.000	ns	b	ab	a	a			
SOD	Ns	0.000	ns	–	–	–	–			
GPX	Ns	0.000	ns	–	–	–	–			

Values presented as means (n = 9 for liver and n = 6 for intestine) and pooled standard error of the mean (SEM). Enzyme activities expressed as mU mg protein^−1^ for G6PDH, GR, and GPX and as U mg protein^−1^ for CAT and SOD

Two-way ANOVA: ns: non-significant (*P* ≥ 0.05).

Liver and intestinal glutathione and oxidative stress index (OSI) were not affected by dietary treatments (Table 1). Oxidized glutathione (GSSG) and OSI values were higher while reduced glutathione (GSH) was lower in the intestine compared to liver. However, total glutathione (tGSH) was similar in both tissues. Further, LPO was higher in the intestine than in the liver and in fish fed diets C and D than diets A and B (Table 1).

Regarding antioxidant enzymes, no organ × diet interaction was observed (Table 2). G6PDH and GPX activities were higher in the liver than in the intestine, while the opposite was true for CAT, GR, and SOD activities. Regarding dietary effects,

GR activity was higher in fish fed diet A than diets C and D, whereas G6PDH, SOD, and GPX activities were unaffected by dietary treatments. For CAT, although Anova indicates a significant dietary effect, that effect was not detected by the Tukey’s multiple range test.

### Distal intestine histological evaluation

**Figure 1:**
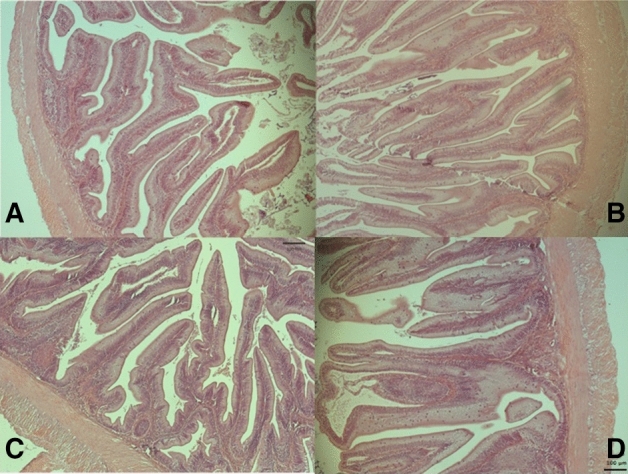
Histomorphology features of distal intestine of gilthead sea bream juveniles fed the experimental diets (**A**–**D**) recorded with Zen software (Blue edition). Normal height of mucosal folds, normal width lamina propria with normal leukocyte infiltration of the lamina propria and submucosa. No signs of inflammation. H-E staining.

Distal intestine histomorphology parameters analyzed, namely enterocyte vacuolization, height of mucosal folds, width of lamina propria and leukocyte infiltration of the lamina propria and submucosa were not affected by dietary treatments (Table 3; Fig. 1).

**Table 3:** Score-based histological evaluation of the distal intestine in gilthead sea bream juveniles fed the experimental diets Values presented as means (n = 6) and pooled standard error of the mean (SEM).

Diets	A	B	C	D	SEM
	2.0/0.2/0.1	1.0/0.4/0.4	0/0.6/0.6	0/0.3/1.5	
Fold height	2.7	1.8	2.3	2.5	0.13
Lamina propria	2.5	2.2	1.7	2.7	0.17
Submucosa	1.0	1.0	1.0	1.0	0.00
Intraepithelial leucocytes	1.8	2.2	2.7	2.3	0.17
Enterocytes	1.5	2.0	1.7	2.8	0.26
Mean score	1.9	1.8	1.9	2.3	0.08

Absence of superscript letters indicates no significant differences between dietary treatments (*P* > 0.05).

### Intestinal microbiota

Number of OTU’s, species richness, in both digesta and mucosa, and digesta diversity were not affected by dietary treatments, except for species diversity in the intestinal mucosa of fish fed diet C which was lower than in fish fed diet A (Table 4).

**Table 4:** Ecological parameters obtained from PCR-DGGE fingerprints of the intestinal allochthonous (digesta) and autochthonous (mucosa) microbiota of gilthead sea bream fed with the experimental diets.

Diets	Digesta					Mucosa				
	A	B	C	D	SEM	A	B	C	D	SEM
	2.0/0.2/0.1	1.0/0.4/0.4	0/0.6/0.6	0/0.3/1.5		2.0/0.2/0.1	1.0/0.4/0.4	0/0.6/0.6	0/0.3/1.5	
OTUs^1^	21.0	22.0	17.7	20.7	0.64	28.7	25.0	22.0	24.0	1.07
Richness^2^	1.2	1.3	1.0	1.2	0.04	1.7	1.5	1.3	1.4	0.06
Diversity^3^	2.9	3.0	2.8	2.9	0.03	3.3^b^	3.2^ab^	3.0^a^	3.1^ab^	0.05
Similarity (%)^4^	80.7	85.8	77.8	85.4	1.37	83.9	76.6	73.2	83.5	2.31

Values presented as means (n = 3) and pooled standard error of the mean (SEM). Different superscript letters indicate significant differences between dietary treatments (*P* < 0.05).

^1^OTUs: Average number of operational taxonomic units.

^2^Margalef species richness: d = (S − 1)/log(N) where S is the number of species, and N is the total number of individuals in the sample.

^3^Shannons diversity index: H’ = − ∑(pi(lnpi)) where pi is the proportion of individuals belonging to the ith species present in the sample.

^4^SIMPER, similarity percentage within group replicates.

**Figure 2:**
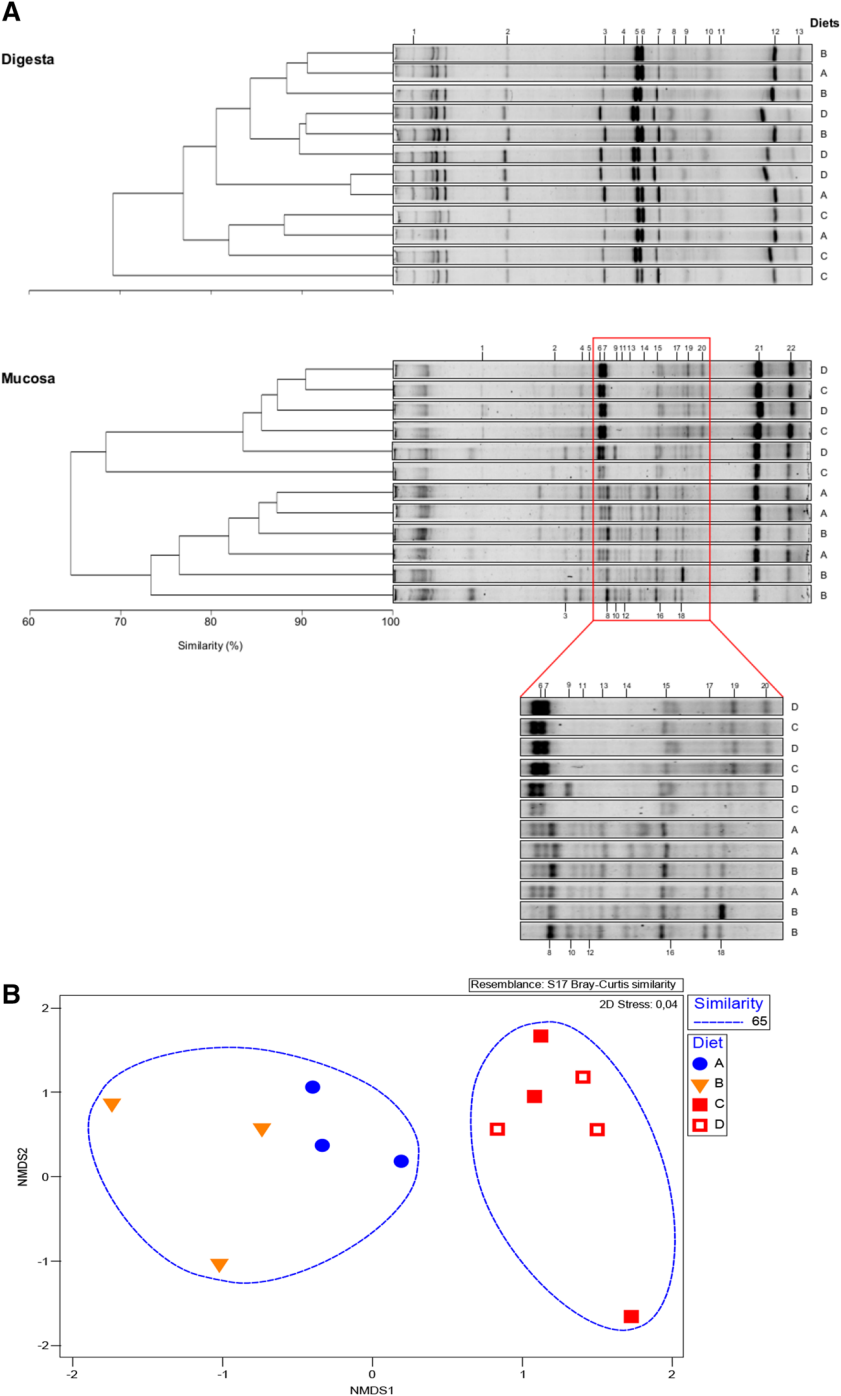
**A** Dendrograms and PCR-DGGE fingerprints of the allochthonous (digesta) and autochthonous (mucosa) intestinal microbiota of gilthead sea bream fed the experimental diets. Numbers (1 to 13 on digesta; 1 to 22 on mucosa) indicate bands excised for sequence analysis, identified on Table 5. **B** Multidimensional scaling (MDS) plot of DGGE bands presence and abundances in intestinal mucosa samples from gilthead sea bream juveniles fed the same experimental diets depicted in A: Diet A ( ); Diet B ( ); Diet C ( ); Diet D ( ). Dotted lines represent 65% similarity between samples.

The Bray–Curtis dendrogram showed that for digesta samples the three dietary replicates failed to cluster (Fig. 2A). For mucosa samples, dendrogram showed that diets with and without ARA supplementation cluster separately. A clearer visualization of this result can be seen in the MDS plot of DGGE bands (Fig. 2B).

Identification of the selected DGGE bands from the digesta and mucosa microbiota is shown in Table 5. Sequence analysis of 35 DGGE bands showed 20 sequences below 97% of sequence similarity, indicating a poor resolution for species identification. However, at the genus level identification, sequence analysis revealed that the dominant allochthonous (digesta) bacterial genus belonged to the Proteobacteria phylum, with three bands of *Klebsiella* genus detected, two bands of *Pantoe*a, and one of *Nitrosospira*. Three bands of *Lactobacillus* (phylum Firmicutes) were also identified.

**Table 5:** Closest relatives (BLAST) to the sequenced PCR-DGGE gel bands (from Fig. 2) of the intestinal communities of gilthead sea bream fed the experimental diets.

Band	Closest known species (BLAST)	Phylum	ID (%)	Accession nr
**Digesta**				
1	*Lactobacillus delbrueckii*	Firmicutes	89	MF784102.1
2	*Lactobacillus aviarius*	Firmicutes	99	LC071826.1
3	*Lactobacillus helveticus*	Firmicutes	99	KX951719.1
4	*Pantoea* sp.	Proteobacteria	86	KR029059.1
5	*Klebsiella pneumoniae*	Proteobacteria	100	MG923524.1
6	*Klebsiella* sp.	Proteobacteria	98	KT301470.1
7	Uncultured bacterium	n/a	97	LC031369.1
8	Uncultured bacterium clone OTU5449	n/a	84	KT788917.1
9	*Pantoea* sp.	Proteobacteria	90	KR029059.1
10	*Klebsiella pneumoniae*	Proteobacteria	89	MG923524.1
11	*Nitrosospira* sp.	Proteobacteria	79	AY293079.1
12	Uncultured bacterium clone ac-25 16S rRNA	n/a	92	KY857639.1
13	Klebsiella pneumoniae	Proteobacteria	96	MH266241.1
**Mucosa**				
1	Uncultured bacterium clone SHFH766	n/a	94	KT981153.1
2	*Asinibacterium* sp.	Bacteroidetes	96	KP301113.1
3	*Lactobacillus helveticus*	Firmicutes	98	KX951719.1
4	*Pseudomonas veronii*	Proteobacteria	96	Kt302366.1
5	*Thalassomonas* sp.	Proteobacteria	90	MG819691.1
6	*Klebsiella pneumoniae*	Proteobacteria	99	MH150802.1
7	*Klebsiella pneumoniae*	Proteobacteria	97	KU550763.1
8	*Janthinobacterium sp.*	Proteobacteria	91	AB196254.1
9	*Bacillus* sp.	Firmicutes	98	FJ195793.1
10	*Thiohalophilus* sp*.*	Proteobacteria	81	KX599116.1
11	*Massilia* sp.	Proteobacteria	97	KY912083.1
12	*Massilia* sp.	Proteobacteria	93	FN386762.1
13	*Edwardsiella tarda*	Proteobacteria	96	MF034724.1
14	*Bacillus* sp.	Firmicutes	78	JX191913.1
15	*Ralstonia* sp.	Proteobacteria	95	MG238553.1
16	*Klebsiella* sp.	Proteobacteria	86	KT300944.1
17	*Planktothricoides* sp.	Cyanobacteria	95	JX628815.1
18	*Bacillus halodurans*	Firmicutes	98	MH299852.1
19	*Klebsiella pneumoniae*	Proteobacteria	99	MH266241.1
20	*Klebsiella pneumoniae*	Proteobacteria	98	MH266241.1
21	*Klebsiella pneumoniae*	Proteobacteria	100	CP029226.1
22	*Klebsiella variicola*	Proteobacteria	100	KR092086.1

Autochthonous (mucosa) bacteria analysis revealed one genus of the phylum Bacteroidetes (*Asinibacterium* sp.) and 4 bands of the phylum Firmicutes (1 *Lactobacillus* and 3 bands of the genus *Bacillus*). Nevertheless, the predominant phylum present in the mucosa was Proteobacteria with 15 bands identified belonging to the *Pseudomonas*, *Thalassomonas*, *Klebsiella*, *Janthinobacterium*, *Thiohalophilus*, *Massila*, *Edwardsiella* and, *Ralstonia* genera (Table 5).

## Discussion

The diets in the presented study had different n6/n3 EFA ratios as well as total n-6 and n-3 LC-PUFA content. Thus, Diet A was rich in ARA and had low EPA and DHA levels; diet B had balanced ARA, EPA and DHA levels; diet C had no ARA and was balanced in EPA and DHA; Diet D had no ARA and had high levels of DHA than EPA.

It is well known that susceptibility of fatty acids (FA) to peroxidation increases with unsaturation, as the weak carbon-hydrogen bond of the bis-allylic methylene groups is particularly prone to oxidation^25^. In the present study, liver and intestine TBARS levels, a well-accepted physiological index of tissue LPO, were inversely related to the dietary n-6/n-3 ratio (diets C and D) and this may be related to increased levels of n-3 LC-PUFA in these tissues. Indeed, a positive correlation between LC-PUFA content in different tissues and lipid peroxidation was already demonstrated in several fish species^14,29,31,42^. Even though liver and intestine FA profiles were not determined in the present study, muscle EPA and particularly DHA contents were higher in fish fed diets C and D than the other diets, while ARA content showed only slight variations^41^ and it is expected that the FA concentrations in liver and intestine should follow a similar pattern. Therefore, a lower concentration of EPA and DHA might explain the lower LPO values in ARA rich diets. Previously it was already observed that the degree of unsaturation of dietary FA correlates with the FA unsaturation in tissues^43,44^ as well as its susceptibility to oxidation^14,45^.

The higher LPO content appeared to be related to elevated contents of DHA in diets C and D. One reason for this may relate to the tendency of DHA to accumulate in mitochondrial cardiolipin that would impair electron transport efficiency and increasing ROS production^46^. The fact that ARA and EPA are not incorporated into mitochondrial cardiolipin may explain the lower LPO content in fish fed diets A and B^46^.

The primary defense against oxidative stress includes antioxidant enzymes such as GR, SOD, CAT, and GPX^25^. GR is essential for maintenance of GSH level by reducing GSSG. The activity was higher in fish fed the high ARA, diet A, followed by diet B, while CAT, SOD and GPX activities were not affected in this experiment. This suggests that the glutathione system was important in the modulation of the redox environment of fish in relation to the dietary EFA composition. This is also supported by tGSH and GSH levels, which were higher (though not statistically higher), and the LPO levels, which were lower, in fish fed diets A and B than the other diets. Similar to the present results, other authors have also reported that higher dietary ARA levels improved the response of antioxidant enzymes in fish^32,33,47^.

As already reported in gilthead sea bream juveniles^48,49^ the antioxidant defense mechanism responded differently in liver and intestine. CAT, GR, SOD, and GSSG were higher in the intestine, while G6PDH and GPX activities were higher in the liver. Overall, LPO values were also considerably higher in the intestine than in the liver, which agrees with previously published data^48^. Higher intestine LPO and OSI values were expected due to the high enterocyte turnover rate that increases susceptibility to oxidation.

In the present study, the dietary effect on intestine histomorphology was accessed in the distal section as previous studies showed that this intestine portion is more sensitive to dietary treatments than the anterior and mid-intestine^50,51^. However, distal intestine histomorphology was not affected by the different dietary n-6/n-3 LC-PUFA levels used in the experimental diets. This confirms previous studies on mid- or distal- intestines of European seabass fed diets with 60–70% of VO blends^52,53^. Also, gilthead sea bream juveniles and Atlantic salmon fed diets with wild type or genetically modified Camelina oil (richer in ARA, EPA, and DHA) showed no differences in distal intestine morphology^54–56^. On the contrary, an accumulation of enterocytes lipid droplets with dietary VO incorporation, leading to lower dietary content of ARA, EPA, and DHA was reported in gilthead sea bream juveniles^57^.

The dietary ARA, DHA, and EPA ratios used in this study, altered gilthead sea bream intestinal microbiota at the mucosal level. The MDS plot and the Bray–Curtis dendrogram representation of mucosal data showed a higher similarity between microbial communities of fish fed diets with elevated levels of EPA/DHA(diets C and D) than within fish fed diets with high ARA content (diets A and B). This shows the potential of dietary fatty acids to select for bacterial species affecting attachment sites of the intestinal mucosa resulting in establishment of different microbial communities^24^. In fact, the isolation of the genus *Thalassomonas* spp., *Janthinobacterium* spp., *Thiohalophilus* spp. and *Massilia* spp. were only isolated in diets A and B. Also, diet C contained 1.2% of EPA + DHA showed lower microbial diversity (Shannon index) at the intestinal mucosa than the other diets. Previous studies reported that 58% and 84% substitution of dietary FO by VO lowered Shannon’s diversity index in gilthead sea bream juveniles^59^. Similarly, other PUFA, such as 2.5% of LA (18:2n-6), were also shown to reduce the culturable intestinal microbiota diversity of Arctic charr (*Salvelinus alpinus*)^60^. On the other hand, the dietary inclusion of 0.5% 1-monoglycerides of short- and medium-chain FA (from C3 to C12) did not influence the same diversity index in gilthead sea bream juveniles^61^. The mechanism for dietary PUFA to reduce fish gut microbiota is not fully understood. It is however interesting to note that reduced bacterial diversity also seems to occur in humans with increase intake of n-3 LC-PUFA^40^. Here the reduction of gut microbial diversity was accompanied by an increase of potential butyrate-producing bacterial species (e.g., *Asinibacterium* sp.), a short-chain fatty acid (SCFA) known to promote gastrointestinal health and host immunity^40^. Accordingly, in Arctic charr fed casein-based diets supplemented with LA, ALA (18:3n-3), or EPA and DHA, the frequency of lactic acid bacteria (LAB, commonly used as probiotics and associated with benefits to host), was higher in diets supplemented with 7% of ALA or 4% of EPA + DHA^58^. Despite this, in our study, the main SCFA-producing species such as *Bacillus* spp. and *Lactobacillus* spp. (LAB)^62^ were present in both digesta and mucosa samples without significant changes between diets.

As reported for other fish species^36^, Proteobacteria and Firmicutes were the dominant phyla in both digesta and mucosa microbiota of gilthead sea bream juveniles in this study, and appear to be predominant in many fish species regardless of dietary treatment^59,61,63–65^. While digesta microbial community is considered transient, mucosal community is assumed to be more stable^36^. The colonization of gut mucosal epithelium is essential for establishing health-promoting bacterial species as autochthonous microbiota and, as consequence, reduce the establishment of opportunistic bacteria^36,37^. The presence of pathogenic species such as *Klebsiella spp.*^66^, despite the different intensity of the bands, was equally distributed among all dietary treatments, and previously isolated from different fish species, suggesting that these microorganisms are part of normal fish microbiota^67–69^ or an opportunistic pathogen attacking stressed animals^70^.

The isolation of one bacterial species from Bacteroidetes phylum (*Asinibacterium* sp.) in diets C and D might highlight the influence of n-3 LC-PUFA in autochthonous microbiota. Although *Asinibacterium* sp. is a recently described genus^71^ whose metabolic potential is not yet totally known, bacteria from Bacteroidetes phylum are also (adding to the Firmicutes, in particular *Bacillus* spp. and *Lactobacillus* spp.^62^), related to the fermentation of dietary non-starch polysaccharides, starch, and sugars into SCFAs as acetate and propionate^72^. These SCFA are absorbed and used as energy sources by the enterocytes and have anti-inflammatory and bactericidal activities^61^. In fact, the isolation of a fish-pathogen such as *Edwardsiella tarda,* causative agent of edwardsiellosis with severe losses in aquaculture^73^, in fish fed with lower levels of n-3 LC-PUFA (diets A and B) might be another indication of the positive effect n-3 LCPUFA had on gilthead sea bream mucosal microbiota.

Increasing bactericidal activity was already associated with long carbon chain length and degree of unsaturation of dietary FA^58,74^. Thus, the higher content of n-3 LC-PUFA might have limited the presence of pathogens such as *E. tarda* in the mucosal microbial community of fish fed diets C and D. Accordingly, a very recent study with golden pompano (*Trachinotus ovatus*) showed that FO substitution by VO increased the abundance of intestinal pathogenic bacteria such as *Mycoplasma* and *Vibrio*^75^. As recently reviewed^76^, gut microbial community, host immune system, and dietary n3-PUFA are interdependent pieces that together control intestinal wall integrity, and thus pathogen proliferation. Furthermore, the host-derived ROS was previously associated with bactericidal activity against pathogens such as *Salmonella* in mice^77^. Also, ROS was associated with gut epithelial response to microbial signals and stimulating immune responses against bacteria^78^. Thus, more studies are required to confirm if increasing ROS production may impair pathogenic bacterial proliferation as it was reported in the present study for fish fed diets C and D.

We report significant modifications in the oxidative status and mucosa microbiota with different dietary n-6/n-3 LC-PUFA content, revealing the importance of correct EFA ratios for gilthead sea bream health. This is especially important with increasing VO incorporation in aquafeeds. The impairment of mucosa colonization by pathogenic bacteria reduces the risk of fish infection and consequently the need for antibiotics utilization, leading to a better image of fish produced in aquaculture to the consumers.

## Material and methods

### Diets composition

Four plant-based diets were formulated as described in Magalhães et al.^41^. Shortly, the experimental diets were isoproteic (47% crude protein; 74% protein from plant feedstuffs and 26% protein from fish meal) and isolipidic (18% crude lipids) with a vegetable oil blend (20:50:30 rapeseed, linseed, and palm oils) as main lipid source. *Mortierella alpine* oil (Vevodar, DSM Food Specialties, the Netherlands); krill oil (*Euphausia superba;* SuperbaKrill Oil, Solchem); and tuna oil (70% DHA; BrudyTechnology) were used to adjust the ARA, EPA, and DHA levels of the diets, to obtain a final ARA/EPA/DHA level of 2%:0.2%:0.1% (Diet A); 1.0%:0.4%:0.4% (Diet B); 0%:0.6%:0.6% (Diet C); 0%:0.3%:1.5% (Diet D). The fatty acids profile of the oils was confirmed before diets were formulated. Ingredients, proximate composition and fatty acids composition of the experimental diets are presented in supplementary file 1—Table S1, and dietary FA composition is presented in additional file 1—Table S2.

## Experimental trial

The growth trial was conducted in CIIMAR, Matosinhos, Portugal. Gilthead sea bream (*Sparus aurata*) juveniles were obtained from a commercial fish farm (Maresa S.A., Ayamonte, Huelva, Spain). Fish were moved to the experimental system after a quarantine period of 1 month and allowed to adapt to the experimental conditions for 15 days. The trial was conducted in a thermo-regulated recirculating marine water system (23.0 ± 1.0 °C; 35 ± 1 g L^−1^ salinity; 7 mg L^−1^ oxygen) equipped with 12 cylindrical fiberglass tanks. Tanks (100 L water capacity) were supplied with filtered seawater (flow of 2.5–3.5 L min^−1^) and kept under controlled photoperiod (12:12 h of light:dark). At the beginning of the experiment, a total of 240 gilthead sea bream juveniles with an initial mean body weight of 15 g were utilized and groups of 20 fish were distributed into the 12 experimental tanks. Each experimental diet was randomly assigned to triplicate groups. The trial was carried on during 56 days and fish were fed by hand, until apparent visual satiation, two times a day, 6 days a week. Extreme care was taken to minimize feed waste.

### Sampling

At the end of the growth trial, fish were randomly sampled 4 h after the morning meal, euthanized by decapitation and dissected on chilled trays. The adjacent adipose and connective tissues of the intestine was removed from 2 fish per tank, and a small portion of the distal intestine (DI, differentiated by a darker mucosa and enlarged diameter from the mid intestine) was collected for histomorphology assessment. Therefore, DI samples were cleaned in phosphate-buffered saline (PBS), carefully absorbed with a paper towel and promptly fixed in phosphate-buffered formalin (4%, pH 7.4) for 24 h and immediately changed to ethanol (70%) until further processing according to Couto et al.^51^. The rest of the intestine was stored at − 80 °C until quantification of oxidative stress enzymatic and non-enzymatic indicators and lipid peroxidation levels. Liver from 3 fish was also sampled for the same purpose. Two other fish per tank were sampled under aseptic conditions (working with an open flame using sterilized solutions, collecting tubes and tools) for allochthonous (digesta) and autochthonous (mucosa) microbiota characterization. Mucosa samples were collected by scraping the internal intestinal mucosal surface, after opening the intestine in its longitudinal axis. Digesta samples were obtained by squeezing the entire intestinal content into a sterile tube. Samples were instantly frozen with liquid nitrogen and stored at − 80 °C until analyzed.

### Chemical analysis

The Association of Official Analytical Chemists methods AOAC^79^ were used to perform the chemical analysis of experimental diets. Dietary starch was quantified as described by Beutler^80^; The Folch et al.^81^ method was utilized for total lipids determination using dichloromethane instead of chloroform. FA methyl esters of oil ingredients and diets were prepared by transmethylation of total lipids extract and analyzed by chromatography using Shimadzu GC-2010 Plus gas chromatograph (Shimadzu Europe GmbH, Germany) equipped with a flame-ionization detector (GC-FID) and an Omegawax 250 capillary column (30 m × 0.25 mm i.d. × 0.25 µm film thickness; Supelco, Bellefonte, USA). FA were identified by comparison with known standard mixtures as described by Magalhães et al.^41^.

### Enzyme activity

Liver and intestine samples were homogenized (dilution 1:7 and 1:5, respectively) in ice-cold 100 mM Tris–HCl buffer, containing 0.1 mM EDTA and 0.1% (v/v) Triton X-100, pH 7.8. All the procedures were performed on ice. Homogenates were centrifuged at 30,000×*g* for 30 min at 4 ºC and supernatants were divided into aliquots and stored at − 80 ºC until use. All assays were carried out at 37 °C in a Multiskan GO microplate reader (Model 5111 9200; Thermo Scientific, Nanjing, China). The specific assay conditions for each enzyme were as follows:

Glucose-6-phosphate dehydrogenase (G6PDH; EC 1.1.1.49) activity was analyzed by measuring the reduction of NADP^+^^82^.

Superoxide dismutase (SOD; EC 1.15.1.1) activity was measured by the ferricytochrome method using xanthine/xanthine oxidase as the source of superoxide radicals^83^. One unit of activity was defined as the amount of enzyme necessary to produce a 50% inhibition of the ferricytochrome c reduction rate.

Catalase (CAT; EC 1.11.1.6) activity was assessed by measuring the decrease of hydrogen peroxide concentration^84^.

Glutathione peroxidase (GPX; EC 1.11.1.9) activity was determined by measuring the NADPH consumption rate generated by the oxidized glutathione (GSSG) produced by GPX activity and reduced by glutathione reductase (GR)^85^.

Glutathione reductase (GR; EC 1.6.4.2) activity was analyzed by measuring the oxidation of NADPH, according to Morales et al.^86^.

Soluble protein concentration was determined according to Bradford^87^ using Sigma-Aldrich protein assay kit and bovine serum albumin as standard.

Except for SOD and CAT which are expressed as units per mg of soluble protein, the activities of the other enzymes are expressed as milliunits per mg of soluble protein. One unit of the enzyme was defined as the amount of enzyme required to transform 1 mmol of substrate per min under the assay conditions.

### Lipid peroxidation

Malondialdehyde (MDA) concentration was used as a marker of lipid peroxidation (LPO) level in the liver and intestine following the methodology described by Buege and Aust^88^. In the presence of thiobarbituric acid, MDA reacts producing colored thiobarbituric acid reacting substances (TBARS) that were measured using a spectrophotometer at 535 nm. Results were calculated from an MDA calibration curve.

### Total and oxidized glutathione

Liver and intestine samples were homogenized (1:10 and 1:5, respectively) in ice-cold solution containing 1.3% 5-sulfosalicylic acid (w/v) and 10 mM HCl, and the whole procedure was done in ice to avoid glutathione oxidation. Homogenates were centrifuged at 14,000*g* for 10 min at 4 °C and the supernatants stored at − 80 °C. Total glutathione (tGSH) and oxidized glutathione (GSSG) were determined accordingly Griffith^89^and Vandeputte et al.^90^ with some modifications according to Castro et al.^14^. Standard curves of reduced glutathione (GSH) and GSSG were used for tGSH and GSSG calculations, respectively. GSH level was calculated by subtracting GSSG from tGSH values.

### Histological processing and morphological evaluation

Distal intestine samples were processed and sectioned using standard histological techniques and stained with hematoxylin and eosin. Blind evaluation of histological preparations was performed, analyzing mucosal folds height, width, and cellularity of the lamina propria and submucosa, number of intraepithelial lymphocytes, nucleus position size, and variation of enterocyte vacuolization^91,92^. A scale scoring system ranging from 1 (normal) to 5 (highly modified) was used as described in Penn et al.^93^. The overall score of histomorphology alterations was calculated by averaging scores of all the parameters. Images were acquired with Zen software (Blue edition; Zeiss, Jena, Germany).

### Microbial diversity

Samples of 2 fish per tank were pooled to reduce variability. The extraction of bacterial DNA from fish intestinal digesta and mucosa was done by weighting around 300 mg of sample to a 2 mL bead-beater (Sigma-Aldrich, Buchs, Switzerland) tube previously prepared with 500 μL STE buffer (0.1 M NaCl, 10 mM Tris, 1 mM EDTA, pH 8) and 0.4 g of glass beads (Sigma-Aldrich G8772). Samples were then homogenized twice for 30 s in the BeadBug bead-beater (Benchmark Scientific, Edison, NJ, USA) at 2500 speed with an interval of at least 30 s on ice. Following 15 min incubation at 75 °C, with gentle agitation every 5 min, tubes were centrifuged for 1 min at 13,000*g* and 500 μL of supernatant was transferred to new sterile 2 mL microcentrifuge tubes. From this point, the protocol used for bacterial DNA extraction was based on the method of Pitcher et al.^94^.

Bacterial 16S rRNA gene fragments were amplified using a touchdown PCR on a T100Thermal Cycler (Bio-Rad Laboratories Lda., Amadora, Portugal), using oligonucleotide primers 16S-358F (which contained a GC clamp at the 5′ end) and 16S-517R^95^. 300 ng of each PCR product was resolved on 8% polyacrylamide gel composed by a denaturing gradient of 40–60% 7 M urea/40% formamide. DGGE was performed using a DCode universal mutation detection system (Bio-Rad Laboratories Lda.) during 16 h at 60 °C, 65 V in 1 × TAE buffer. Gels were stained for 1 h with SYBR-Gold Nucleic Acid Gel Stain (Thermo Fisher Scientific, Waltham, Massachusetts, EUA) and imaged on a Gel Doc EZ System (Bio-Rad Laboratories Lda., Amadora, Portugal). Distinct bands were excised from the gel and eluted in 20 µL ultrapure water prior to DNA re-amplification using the same oligonucleotide primers as above, but without the GC clamp^95^. Amplicons were sequenced to identify microbiota OTUs (Operational Taxonomic Units). Phylogenetic analysis, to identify the closest known species, was done by comparison with sequences in the GenBank non-redundant nucleotide database using BLAST (https://www.ncbi.nlm.nih.gov) (Macrogen Europe, Amsterdam, The Netherlands). Only sequences higher than 100 bp reads and 80–100% query coverage were considered valid identification.

### Statistical analysis

Data are presented as the mean and pooled standard error. Normality and homogeneity of variances were tested by the Shapiro–Wilk and Levene tests, respectively, and normalized when appropriate. Statistical evaluation of the data was done by one-way or two-way ANOVA. When *p *values were significant (*p* < 0.05), the means were compared with Tukey’s multiple range test. Histological data were analysed by the Kruskal–Wallis non-parametric test because the data were neither normal nor homogeneous and could not be normalized. All statistical analyses were performed using SPSS 24.0 software package for Windows (IBM SPSS Statistics, New York, USA).

Microbiota data analysis was done according to Serra et al.^69^ using the DGGE banding patterns, the band's intensity was measured with Quantity One 1-D Analysis Software v4.6.9 (Bio-Rad Laboratories Lda., Amadora, Portugal) and converted into absence/presence matrices. The calculation of the relative similarities between experimental groups and replicates was done with Primer software v7.0.5. 5 (PRIMER-E, Ivybridge, UK). Non-metric multidimensional scaling (MDS) was based on Bray–Curtis similarities using relative band abundances. Data representation from MDS was considered reliable considering the Kruskal stress value (< 0.2)^96^. Species Richness was established with the use of Margalef’s diversity index, while Shannon–Weaver index was utilized to establish species diversity. Similarity percentages (SIMPER) were utilized to represent the relative similarities between studied groups.

### Ethics approval and consent to participate

The experiment was approved by CIIMAR ethical committee for Managing Animal Welfare (ORBEA), in compliance with the European Union directive 2010/63/EU and the Portuguese Law (DL 113/2013).

## Supplementary information

Supplementary Information.

## Data Availability

The data generated during and/or analysed during the current study are available from the corresponding author on reasonable request.
