# Supplementary material for: Oxidative status and intestinal health of gilthead sea bream (Sparus aurata) juveniles fed diets with different ARA/EPA/DHA ratios
Source: Sci Rep. 2020 Aug 14;10:13824. doi: 10.1038/s41598-020-70716-5 (PMC7427802; doi:10.1038/s41598-020-70716-5)
Supplement: Supplementary file 1 — Supplementary Information. [file 41598_2020_70716_MOESM1_ESM.docx]

OXIDATIVE STATUS AND INTESTINAL HEALTH OF GILTHEAD SEA BREAM (*Sparus aurata*) JUVENILES FED DIETS WITH DIFFERENT ARA/EPA/DHA RATIOS

R. Magalhães^1,2^, I. Guerreiro^1^, R.A. Santos^1,2^, F. Coutinho^1^, A. Couto^1,2^, C.R. Serra^1^, R. E. Olsen^3^, H. Peres^1,2^, A. Oliva-Teles^1,2^

^1^CIMAR/CIIMAR – Centro Interdisciplinar de Investigação Marinha e Ambiental, Universidade do Porto, Terminal de Cruzeiros do Porto de Leixões, Av. General Norton de Matos, 4450-208 Matosinhos, Portugal

^2^Departamento de Biologia, Faculdade de Ciências, Universidade do Porto, Rua do Campo Alegre, Edifício FC4, 4169-007 Porto, Portugal

^3^Norwegian University of Science and Technology, Department of Biology, Trondheim, Norway

# * ruipedromag10@gmail.com

| **Diets** | **A** | **B** | **C** | **D** |
| --- | --- | --- | --- | --- |
| **(ARA/EPA/DHA ratio)*** | **2.0/0.2/0.1** | **1.0/0.4/0.4** | **0/0.6/0.6** | **0/0.3/1.5** |
| *Ingredients* (% DM) |  |  |  |  |
| Fish meal^1^ | 17.5 | 17.5 | 17.5 | 17.5 |
| Corn gluten meal^2^ | 17.0 | 17.0 | 17.0 | 17.0 |
| Soybean meal^3^ | 17.5 | 17.5 | 17.5 | 17.5 |
| Wheat gluten meal^4^ | 10.5 | 10.5 | 10.5 | 10.5 |
| Wheat meal^5^ | 15.4 | 15.4 | 15.4 | 15.4 |
| Taurine^7^ | 0.3 | 0.3 | 0.3 | 0.3 |
| Lysine^8^ | 0.3 | 0.3 | 0.3 | 0.3 |
| Vegetable oil blend^9^ | 10.6 | 11.0 | 10.7 | 12.7 |
| Choline chloride (50%) | 0.5 | 0.5 | 0.5 | 0.5 |
| Mineral premix^10^ | 1.0 | 1.0 | 1.0 | 1.0 |
| Vitamin premix^11^ | 1.0 | 1.0 | 1.0 | 1.0 |
| Binder^12^ | 1.0 | 1.0 | 1.0 | 1.0 |
| CaHPO_4_ | 3.3 | 3.3 | 3.3 | 3.3 |
| VEVODAR^13^ | 4.2 | 2.1 | ― | ― |
| EFA mix^14^ | ― | 1.7 | 4.1 | ― |
| DHA 70% Algatrium^15^ | ― | ― | ― | 2.1 |
| *Proximate analyses (% dry weight)* |  |  |  |  |
| Dry matter (%) | 89.6 | 90.6 | 87.6 | 88.4 |
| Crude Protein | 46.5 | 46.9 | 47.4 | 47.8 |
| Crude Lipid | 18.7 | 17.8 | 18.3 | 19.1 |
| Ash | 7.4 | 8.0 | 7.6 | 7.7 |
| Starch | 13.1 | 10.3 | 10.3 | 11.2 |
| Gross energy (kJ g^-1^) | 21.9 | 21.5 | 21.5 | 21.4 |

**Table S1**. Ingredient and proximate analysis of experimental diets.

CP: Crude protein; CL: Crude lipids; DM: Dry matter.

^1^Steam Dried LT-FM, Pesquera Centinela, Chile (CP: 69.7%; CL 7.2%).

^2^Sorgal, S.A. Ovar, Portugal (CP: 70.7%; CL: 3.6%).

^3^Sorgal, S.A. Ovar, Portugal (CP: 51.2%; CL: 3.7%).

^4^Sorgal, S.A. Ovar, Portugal (CP: 86.2%; CL: 3%).

^6^Sorgal, S.A. Ovar, Portugal. (CP: 14.5%; CL: 2.4%).

^7^Feed-grade taurine, Sorgal, S.A. Ovar, Portugal.
^8^Feed-grade lysine, Sorgal, S.A. Ovar, Portugal.
^9^Vegetable oil blend: 20% rapeseed oil + 50% linseed oil + 30% palm oil

^10^Minerals (mg kg^-1^ diet): cobalt sulphate, 1.91; copper sulphate, 19.6; iron sulphate, 200; sodium fluoride, 2.21; potassium iodide, 0.78; magnesium oxide, 830; manganese oxide, 26; sodium selenite, 0.66; zinc oxide, 37.5; dicalcium phosphate, 8.02 (g kg^-1^ diet); potassium chloride, 1.15 (g kg^-1^ diet); sodium chloride, 0.4 (g kg^-1^ diet).

^11^Vitamins (mg kg^-1^ diet): retinol, 18000 (IU kg^-1^ diet); calciferol, 2000 (IU kg^-1^ diet); alpha tocopherol, 35; menadion sodium bis., 10; thiamin, 15; riboflavin, 25; Ca pantothenate, 50; nicotinic acid, 200; pyridoxine, 5; folic acid, 10; cyanocobalamin, 0.02; biotin, 1.5; ascorbyl monophosphate, 50; inositol, 400.

^12^Carboxymethylcellulose sodium. Sigma-Aldrich Química, Portugal

^13^DSM Nutritional Produtcs (ARA: 47.4 %FA;EPA: 0.11 %FA;DHA: 0.04 %FA)

^14^85% Krill oil (SuperbaKrill^TM^ Oil, Solchem; ARA:0.78 %FA; EPA:21.1 %FA; DHA: 11 %FA) + 15% DHA

^15^BrudyTechnology (Tuna oil; ARA: 0 %FA; EPA: 10.2%FA; DHA: 89.5 %FA)

*ARA/EPA/DHA ratio expresses as % DM

| Diets | A | B | C | D |  |
| --- | --- | --- | --- | --- | --- |
| (ARA/EPA/DHA ratio) | **2.0/0.2/0.1** | **1.0/0.4/0.4** | **0/0.6/0.6** | **0/0.3/1.5** |  |
| SFA |  |  |  |  |  |
| 10:0 | 0.04 | 0.05 | 0.05 | 0.04 |  |
| 12:0 | 0.08 | 0.08 | 0.10 | 0.07 |  |
| 14:0 | 0.84 | 1.45 | 2.48 | 0.79 |  |
| 15:0 | 0.09 | 0.10 | 0.14 | 0.07 |  |
| 16:0 | 15.0 | 16.3 | 18.1 | 15.7 |  |
| 17:0 | 0.15 | 0.14 | 0.13 | 0.12 |  |
| 18:0 | 3.99 | 3.51 | 3.06 | 3.04 |  |
| 20:0 | 0.43 | 0.35 | 0.28 | 0.28 |  |
| 22:0 | 0.85 | 0.53 | 0.21 | 0.18 |  |
| 24:0 | 2.66 | 1.44 | 0.16 | 0.15 |  |
| MUFA |  |  |  |  |  |
| 16:1i | 0.06 | 0.07 | 0.09 | 0.06 |  |
| 16:1n-7 | 0.80 | 1.17 | 1.77 | 0.77 |  |
| 18:1n-9 | 23.2 | 24.5 | 25.7 | 26.2 |  |
| 18:1n-7 | 1.11 | 1.50 | 2.11 | 1.19 |  |
| 20:1n-11 | 0.05 | 0.05 | 0.05 | 0.03 |  |
| 20:1n-9 | 0.42 | 0.40 | 0.40 | 0.33 |  |
| 22:1n-11 | 0.19 | 0.19 | 0.21 | 0.17 |  |
| 22:1n-9 | 0.05 | 0.08 | 0.12 | 0.03 |  |
| 24:1n-9 | 0.19 | 0.17 | 0.16 | 0.12 |  |
| PUFA |  |  |  |  |  |
| 16:2n-4 | 0.07 | 0.10 | 0.15 | 0.07 |  |
| 16:3n-4 | 0.06 | 0.07 | 0.08 | 0.06 |  |
| 16:4n-1 | 0.12 | 0.16 | 0.22 | 0.12 |  |
| 18:2n-6 (LA) | 16.1 | 16.27 | 15.8 | 16.5 |  |
| 18:3n-6 | 0.52 | 0.28 | 0.04 | 0.01 |  |
| 18:3n-3 (ALA) | 17.0 | 18.1 | 17.5 | 20.5 |  |
| 18:4n-3 | 0.12 | 0.28 | 0.48 | 0.11 |  |
| 20:2n-6 | 0.12 | 0.08 | 0.05 | 0.04 |  |
| 20:3n-6 | 0.94 | 0.49 | 0.04 | 0.02 |  |
| 20:4n-6 (ARA) | 10.6 | 5.43 | 0.18 | 0.11 |  |
| 20:4n-3 | 0.05 | 0.07 | 0.10 | 0.05 |  |
| 20:5n-3 (EPA) | 0.96 | 2.02 | 3.24 | 1.70 |  |
| 21:5n-3 | 0.04 | 0.12 | 0.21 | 0.50 |  |
| 22:4n-6 | 0.08 | 0.05 | 0.02 | 0.01 |  |
| 22:5n-3 | 0.12 | 0.29 | 0.49 | 1.33 |  |
| 22:6n-3 (DHA) | 0.70 | 2.06 | 3.52 | 7.71 | |
| ∑SFA | 24.2 | 24.1 | 24.9 | 20.6 |  |
| ∑ MUFA | 26.2 | 28.2 | 30.8 | 28.9 |  |
| ∑ PUFA | 47.7 | 46.0 | 42.2 | 48.8 |  |
| ∑n-6 | 28.4 | 22.6 | 16.2 | 16.6 |  |
| ∑n-3 | 19.0 | 23.0 | 25.5 | 31.9 |  |
| n-6/n-3 | 1.50 | 0.98 | 0.63 | 0.52 |  |

**Table S2.** Fatty acid composition of the experimental diets (% of total fatty acids).
FA: fatty acid; LA: Linoleic acid; ALA: α-Linolenic acid; MUFA: monounsaturated fatty acid; PUFA: polyunsaturated fatty acid; SFA: saturated fatty acid.

**RAW DATA** of

**Submission ID 2d9fe874-3fec-4f8a-b835-cf7a8e308bb1**

" OXIDATIVE STATUS AND INTESTINAL HEALTH OF GILTHEAD SEA BREAM (Sparus aurata) JUVENILES FED DIETS WITH DIFFERENT ARA/EPA/DHA RATIOS" **by Magalhães *et al.***

1. **Original DGGE gels**

1. **Original Data from Bands Signal Quantification using Quantity One (Bio-Rad)**
